# I Know Some People: The Association of Social Capital With Primary Health Care Utilization of Residents in China

**DOI:** 10.3389/fpubh.2021.689765

**Published:** 2021-07-30

**Authors:** Weiwei Zhang, Yuankai Huang, Mengqing Lu, Guohua Lin, Tian Wo, Xiaoyu Xi

**Affiliations:** The Research Center of National Drug Policy & Ecosystem, China Pharmaceutical University, Nanjing City, China

**Keywords:** primary health care, utilization, social capital, multilevel analysis, China

## Abstract

**Background:** Primary health care (PHC) services are underused due to the unbalanced distribution of medical resources. This is especially true in developing countries where the construction of PHC systems has begun to take effect. Social capital is one of the important factors affecting primary health care utilization.

**Method:** This study investigated the utilization of PHC services by Chinese community residents in the past year. Social capital, PHC utilization, age, health care insurance, etc., were measured. A multilevel negative binomial model was adopted to analyze the association of social capital with PHC utilization.

**Results:** Data of 5,471 residents from 283 communities in China were collected through a questionnaire survey in 2018. The results showed that community social capital (CSC) is significantly associated with PHC utilization in China, but individual social capital (ISC) had no significant association with PHC utilization. A one-standard deviation increase in the CSC leads to a 1.9% increase in PHC utilization. Other factors like gender, education, income, health insurance, health status, etc., are significantly associated with PHC utilization in China.

**Conclusions:** Community social capital plays a more important role in promoting PHC utilization, while ISC plays an unclear role in PHC utilization by the residents of China.

## Introduction

In 1978, the Declaration of Alma-Ata stated the goal of “health for all by the year 2000,” and primary health care (PHC) an essential strategy to achieve this goal, was defined as “scientifically sound and socially acceptable methods and technology made universally accessible to individuals in the community through their full participation” (1). Since then, great importance was given to the development of PHC globally, and efforts were made by countries to incorporate PHC into their health system, through which the equity of accessibility and universal coverage of health could be achieved (2). There exists indications that a well-developed PHC system facilitates the utilization of PHC by residents, which refers to the extent of residents' usage of services provided by PHC institutions (3). Specifically, effective PHC utilization could alleviate the undersupply of regional health care services caused by the imbalanced allocation of health resources and improve the overall efficiency of health systems (4). Also, PHC helps patients achieve better health outcomes at relatively low costs (5).

In the past few decades, some developed countries have built sophisticated PHC systems, while the development of PHC systems in developing countries is still in the early stages and PHC utilization of residents was generally insufficient (6), such as that in China. In the current three-tier hierarchical health care system of China, PHC provides generalist clinical care and basic public health services covering the scope of the geographically nearby community and operate a referral system to secondary and tertiary hospitals (7). Different from those in developed countries, a referral from the PHC is nonmandatory for visiting high-level health institutions under the current three-tier health care system of China (8) (see Box 1). However, due to the non-mandatory first diagnosis in a PHC, an enormous number of patients with health problems that could be well-handled by a PHC still directly seek health care in high-level health institutions, which may harm the steady and sufficient utilization of PHC by patients, and eventually lower the revenue of PHC and reduce the practice experience of the primary health professionals and administrators (9). Therefore, in addition to investment increase and policy system perfection, it is necessary for Chinese policymakers to identify the strategies to facilitate PHC utilization from other perspectives.

Box 1 **The current three-tier health care system of China.**

Since China's new health care reform initiated in 2009, the government has been putting effort into the improvement of the availability and affordability of PHC by increasing governmental investment and reinforcing policy support of the development of PHC, aiming at the establishment of three-tier health care network among which the tertiary hospitals provide healthcare for patients with acute and critical illnesses and complicated diseases, secondary hospitals mainly receive patients referred from secondary hospitals who are recovering from acute illnesses, surgery or critical conditions, and primary healthcare institutions provide treatment, rehabilitation and nursing services to patients with clearly diagnosed and stabilized health conditions. One of the primary goals of building such a network is to enhance the role of gate keeper for PHC by increasing the utilization of PHC institutions.

There exist several studies on the factors of PHC utilization. Many studies focused on epidemiological factors like chronic or severe diseases (10–12). Economic factors like the expenditures of primary services (13) and family income (14) also affect PHC utilization. However, several recent studies showed that social factors may have an indispensable impact on PHC utilization. For example, Adekanmbi et al. (15) found that people with higher education have lower utilization of PHC. According to Xiaoxin et al. (16), women, older age groups, and low-income groups are more inclined to visit PHC. Although social factors have drawn increasing attention, social capital, which is an important social attribute of the residents and has theoretical relation to health-service utilization, still lacks study in its relation to PCH utilization, especially in developing countries.

Social capital is a connotative concept, and the definitions vary in disparate disciplines. In this study, the widely accepted definition adopted in the public health area is used: networks, groups, or relationships between people, based on mutual trust, a set of norms, and understanding that are formed to facilitate collective action for common benefits (17). Social capital is considered to be closely related to a specific cultural background and research perspective. Also, social capital affects individual behavioral characteristics in specific cultural contexts, so cultural differences may affect the results of social capital research. For example, as a typical Asian collectivistic country, China has the highest level of collectivism and interpersonal trust among the 42 countries analyzed in a study that examined the social capital of residents in individualistic countries and collectivistic countries (18). In a society with such strong ties, close group relationships usually emerge among residents and allow them to easily obtain high social capital, which, in China, plays an important role in interpersonal interactions and imperceptibly affects all aspects of their daily lives, including health-related behaviors (19).

Moreover, it is important to distinguish individual social capital (ISC) from the social capital of the community level because of its different mechanisms of influencing the behaviors and its scope of influence (20). Individual social capital reflects social support, social influence, social engagement, and attachment, while community social capital (CSC) can be defined as the density of trust, network, or cooperation within a given community (21). Currently, multiple studies on social capital considered both individual and community levels (22). Multilevel analysis offers excellent tools to improve the understanding of the mechanism through which social capital influences health behavior. However, only limited research studies have emphasized the importance of examining multilevel social capital and its influence on health behaviors (12, 23–25), which makes applying multilevel perspectives and techniques a valuable attempt for future studies.

The impact of social capital on PHC utilization has been extensively validated in developed countries, but only limited evidence was validated from developing countries (26–29). Due to the differences in social culture and health care systems, conclusions reached in developed countries may not be applicable to developing countries (30). For example, substantial cultural difference means that the United States is characterized by individualism that regards trustworthiness as the main evaluating standard, whereas China is characterized by collectivism and focuses more on networking.

China is a developing country, and existing research on health care utilization in China mainly focused on the service competence of health care institutions (31), the type of health insurance (32, 33), and other personal characteristics (34, 35), which makes previous studies quite limited as regards to the impact of social capital on PHC utilization.

**Figure 1:**
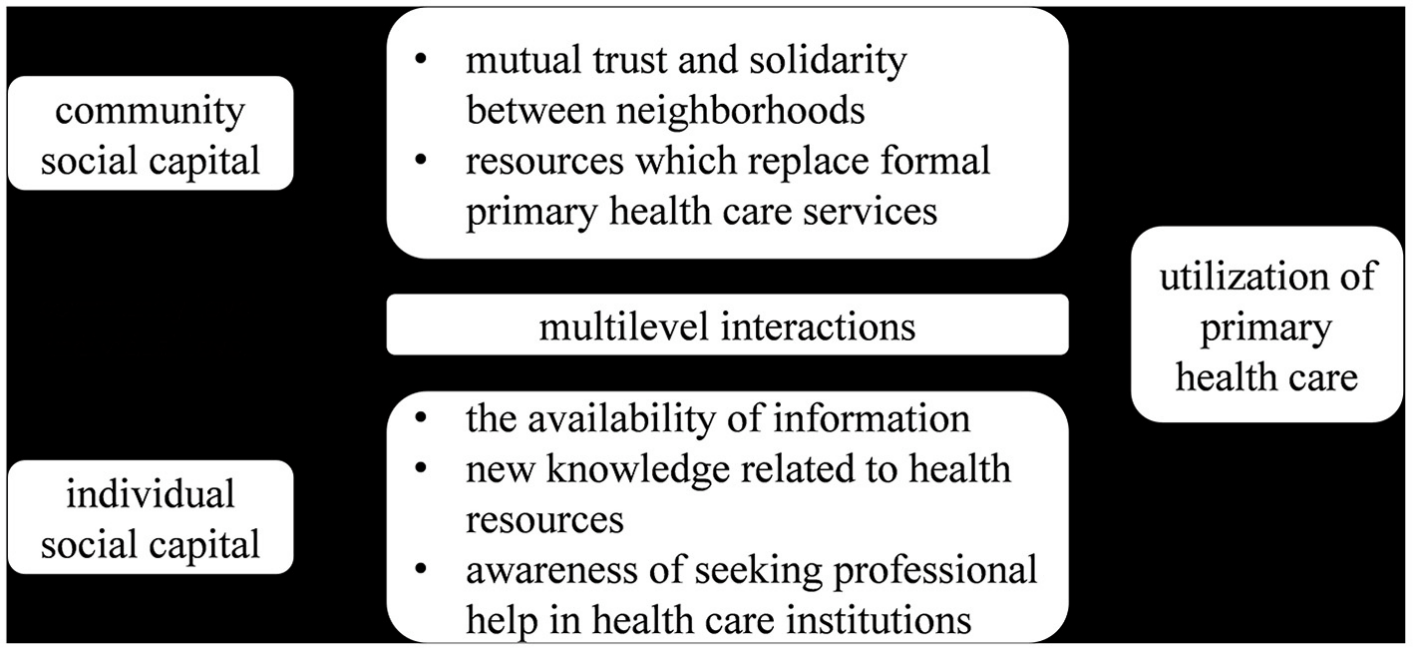
Conceptual model of association of community and individual social capital with PHC utilization.

With studies on countries that share some common features with China, this study assumed that, in China, there are two potential pathways through which social capital affects PHC utilization, as shown in Figure 1. One potential pathway is information networks, through which ISC can facilitate PHC information sharing among individuals in communities (36). In China, many public health services, especially health education, are usually offered in communities, so community organizations, such as PHC institutions, play an important role in health information sharing. Therefore, participation in community activities can help residents to obtain health information or sharing information with other individuals in his/her own social network, so that ISC may promote PHC utilization by increasing the availability of information, providing new knowledge related to health resources, and creating the awareness of residents seeking professional help in health care institutions when health issues occur. The other potential pathway is the norm channel, which represents a common belief (12). CSC can convey a set of attitudes and values of the people through a norm channel, which evokes a sense of mutual trust and solidarity among neighbors that increase the ability of groups to implement and maintain social norms, such as health behavior norms, and because of the universal underutilization of PHC, the norm channel may reduce the utilization of PHC. Additionally, CSC can also reduce the utilization by using resources that replace formal health services. Based on the above two potential pathways, there are possible multilevel interactions between their effects on PHC utilization, which should be identified when evaluating the effects of ISC and CSC (12).

Based on the two theoretical pathways discussed above, this paper aimed to validate the association of social capital with PHC utilization in China and analyze the differences between the association of ISC and CSC with PHC utilization. This paper assumed that higher ISC can increase the utilization of PHC, while higher CSC reduces the utilization of PHC by replacing formal health services. A multilevel regression model was employed to precisely differentiate the association of ISC and CSC with PHC utilization and meanwhile adequately controls the confounders. This study may not only provide new insights into the health policymaking and primary health care (PHC) development targeting on the promotion of PHC utilization in China but also provides references for other developing countries.

## Methods

### Setting and Study Design

A survey covering 30 provincial administrative regions (including provinces, autonomous regions, and municipalities directly under the central government) in mainland China was conducted using a stratified sampling strategy: (1) the primary sampling units (PSUs) include a total of 30 provincial administrative units, and the other four provinces were excluded due to inaccessibility of accessing respondents and data (the excluded provinces account for only 2.46% of the population of China, thus the exclusion had minor influence on the representativeness of the sample among the population of China); (2) the secondary sampling units (SSUs) are cities or counties within each provinces or autonomous regions (for the cases of municipalities, the SSUs are their districts), and the SSUs in each PSU were evenly divided into two “population-groups” by median of their population sizes; (3) SSUs in each “population-group” were then evenly divided into two “PHC-groups” by median of their numbers of PHCs per million population; (4) and then one SSU was sampled by convenience in each “PHC-group,” and a total of 120 SSUs were eventually sampled; (5) within each SSU, three communities were sampled by convenience as tertiary sampling units (TSUs); (6) In each TSU, 20 residents were interviewed through convenience sampling. Above all, a sample of 7,200 respondents from 360 TSUs was expected.

The inclusion criteria of respondents were: (1) 18 years old or older; (2) having resided in the sampled communities for over 3 months; and (3) the respondents visited any health institutions (regardless of the level and type) for outpatient services within 12 months before the survey (Chinese government encourages the residents to take their initial clinical visit in PHCs, but initial visits to higher-level health care institutions are also allowed. The last inclusion criterion was designed so that most choices of respondents of health care institutions they utilized were by their preference but not their needs).

### Data Collection and Quality Control

A total number of 100 undergraduates majoring in public health were recruited and trained as data collectors for performing the survey, and 10 postgraduate students majoring in public health were recruited and trained as survey monitors for checking the errors of questionnaire filling or electronic file damage.

Data collectors were assigned to survey the teams by pairs, and each team performed the survey in one sampled city or a few that were geographically near to each other. Face-to-face interviews were conducted in the survey. The data collectors briefly introduced the aim of the study and the procedure of the survey and checked if the potential respondents, who were willing to participate, met the inclusion criteria. Those who met the criteria were then asked to sign the informed consent with necessary explanation by the data collectors. The interviews were performed in undisturbed public places that are convenient for the respondents. The data collectors accessing the questionnaire with the online survey system on mobile devices orally asked each item of the questionnaire and recorded the responses in the survey system. The data collectors did not provide any perspective related to the contents of the questionnaire to the respondents. The recorded responses were immediately transferred to the survey monitors for checking. Invalid questionnaires were sent back within few minutes for reinterview. A random of 5% of the questionnaires was checked at the end of the survey (37). The responses were automatically converted into electronic data for analysis software.

### Instruments

#### Primary Health Care Utilization

Primary health care utilization is the dependent variable in this research, and it was measured by the number of primary-care visits a person made in a given year. Respondents were asked “how many times have you visited the PHC institutions in the past 12 months?” and were asked to provide an exact number to represent his/her PHC utilization. This was an approach used in several previous studies (38).

#### Social Capital

Social capital is widely regarded as a feature of social organization that includes trust, norms, and social networks (39). Here, trust refers to the expectation based on shared norms generated in a group showing honest, cooperative, and normative behaviors (40), and this is the core idea of social capital. Previous theoretical and practical evidence showed that trust is a very stable measure of social capital and can be used in different countries and regions with perfect predictive validity (41, 42).

In this study, social capital was measured with the concept of trust, which is a frequently used and currently an effective measurement of social capital. The dimensions of “trust and solidarity” (18 items in all) were used in the Integrated Questionnaire for the Measurement of Social Capital (SC-IQ) recommended by the World Bank, which has been designed for developing countries (43). Evidence shows that this instrument can provide useful and abundant information on social capital.

At the community level, we considered a contextual social trust variable aggregated from individual responses to questions on interpersonal trust. That is, the community-level social capital is calculated by weighing the arithmetic average of social capital measured at the individual level but excluding the contribution of each respondent to the mean value (44). The authors assume that respondents living in the same community have the same degree of CSC (45), and the CSC score of each respondent was the mean of the ISC scores of all the respondents from the community that this respondent was from.

### Covariates

This study included household registration (household certificate for individuals issued by local government in China) (rural/urban location), perceived community size (very small/small/fair/large/very large), gender (male/female), education (Junior high school/High school/Junior college /Undergraduate/Graduate student or higher), age, spouse occupation, individual annual non-medical expenditures (Chinese Yen), years lived at current location (1–3 years/4–5 years/6–10 years/11–19 years/≥20 years), renting housing (no/yes), body mass index (BMI), type of health care insurance [urban employee basic medical insurance (UEBMI)/urban resident basic medical insurance (URBMI)/new cooperative medical scheme (NCMS)], commercial medical insurance (no/yes), smoking (no/yes), self-health grade (very good/good /fair/poor/very poor, Chinese communist party (CCP) membership (no/yes), and number of chronic disease (counting variable) as control variables that may have been proven to be factors of health-service utilization in previous studies (46–49). The individual annual non-medical expenditures were used as a proxy for income, and medical expenditures were excluded to avoid potential endogenous effects of PHC utilization and medical expenditures (50).

A pretest was conducted in 11 communities in Hangzhou city in Zhejiang province of China, by which the understandability and readability of the whole questionnaire were tested to be good, and the reliability (Cronbach's Alpha = 0.686) and validity (Kaiser-Meyer-Olkin Measure of Sampling Adequacy = 0.807, Bartlett's Test of Sphericity: Approx. Chi-Square = 1407.759, df = 153, Sig. = 0.000) of the instrument for social capital were tested to be acceptable. The data collection method was also tested to be feasible. For the English version of the questionnaire, see Appendix 1.

### Statistical Analysis

A multilevel model was applied because the interclass correlation coefficient of the dependent variable in the community level was lower than 0.4 and *p*-value of likelihood ratio test result of the model was lower than 0.05. And if so, a multilevel model was used to evaluate the association of ISC, CSC, and covariates with PHC utilization, which may avoid the Robinson effect (51). Three levels were structured. The individual level (level 1), which contained ISC, CSC, and all covariates; the community level (level 2), which only contained CSC; and the city level (level 3), which contained no independent variable. Since the dependent variable in this study is a counting variable, it was linked to the negative binomial model. The cross-level interaction was tested using the interclass correlation coefficient and likelihood ratio test vs. the negative binomial model.

As previous similar studies recommended (52), four models were performed to assess the association of ISC and CSC with PHC utilization in the case of controlling covariates. Model 1 was a null model containing no independent variables, and it estimates the variance for each level before introducing the individual- and community-level independent variables. Only individual variables were added into Model 2, and only CSC was added to Model 3 to assess its effect on PHC utilization. Model 4 included all the variables. Comparing Models 2, 3, and 4 enables us to assess the association of individual and contextual social capital with PHC utilization separately and simultaneously. In the above model, the variables were centralized to avoid estimation errors caused by different dimensions or self-variation (53). Furthermore, by employing the group-mean centering procedure, the authors were able to disentangle the “pure” individual vs. contextual effects of social capital on PHC utilization.

The data were analyzed using SPSS 24 (IBM Corporation, Armonk, NY, USA) and STATA 14.

## Results

Table 1 presents the sociodemographic characteristics of the sample. A total number of 5,471 respondents (dropout rate = 24.0%) from 283 communities (dropout rate = 21.3%) participated in the survey. The mean age was 39.90, among which the ratio of male to female respondents was around 1:1.33. Approximately, 30.9% of the respondents had received an undergraduate education, and this group represented a higher proportion than the respondents with other levels of education. About 7.6% of the spouses of the respondents were unemployed, and most of the employed people were public servants, self-employed entrepreneurs, or workers. In addition, this study conducted research in both urban and rural areas with a ratio of rural to urban respondents of approximately 1:1.15. Most of the urban respondents are insured by the urban employee basic medical insurance (UEBMI) or the urban resident basic medical insurance (URBMI), and URBMI covered more people (36.5%). Most rural respondents are insured by the new cooperative medical scheme (NCMS) (28.8%), and only a small proportion of the respondents (6.0%) said that they are uninsured by any healthcare insurance. The main characteristics of the sample were similar to the population as described in the 2019 national data of China provided by the National Bureau of Statistics of China, including the gender (male = 51.09%), age (0–14 years old: 16.78%; 15–64 years old: 68.58%; over 65 years old: 13.52%), household registration (urban population: 62.71), etc.

**Table 1:** Social demographic statistics and characteristics of respondents.

**Variable**	**N (Populations) = 5,471**	
	**N (Communities) = 283**	
	**Mean/Proportion (S.D.)**	**Range**
PHC Utilization	1.76 (3.12)	0.00–60.00
ISC	58.87 (9.36)	19.00–84.00
CSC	58.79 (5.96)	40.00–81.15
Age	39.91 (14.69)	18.00–89.00
Income	51020.52 (76794.80)	0.00–150000.00
BMI	21.96 (3.18)	12.08–58.82
Chronic Number	0.53 (0.88)	0–9
Gender		
Male	43.0%	
Female	57.0%	
Household registration		
Rural	39.5%	
Urban	60.5%	
CCP membership		
Yes	19.8%	
No	80.2%	
Education		
Elementary school or lower	9.8%	
Junior high school	19.9%	
High school	21.4%	
Junior college	14.4%	
Undergraduate	30.9%	
Graduate student or higher	3.6%	
Marriage		
Single	25.0%	
Married	70.8%	
Divorce or separation	3.2%	
Others	0.9%	
Has children		
Yes	71.3%	
No	28.7%	
Community Scale		
Very small	13.7%	
Small	17.0%	
Fair	47.5%	
Large	18.2%	
Very large	3.6%	
Spouse Occupations		
Public servants	10.8%	
Teacher	6.7%	
Manager	6.5%	
Worker	11.3%	
Farmer	8.6%	
Self-employed entrepreneur	13.7%	
Others	7.8%	
None	7.6%	
Without spouse	27.0%	
Years lived at current location		
<1 year	4.6%	
1–3 years	18.7%	
4–5 years	18.0%	
6–10 years	19.5%	
11–19 years	13.7%	
≥20 years	25.5%	
Rent or home ownership		
Renting	13.8%	
Home Ownership	86.2%	
Healthcare Insurance		
UEBMI	28.7%	
URBMI	36.5%	
NCMS	28.8%	
None	6.0%	
CMI		
Yes	28.8%	
No	71.2%	
Smoke		
Yes	55.1%	
No	44.9%	
Self-health grade		
Very good	1.1%	
Good	6.7%	
Fair	41.7%	
Poor	37.3%	
Very poor	13.1%	

*Household registration - A record that officially identifies area residents; CMI, Commercial Medical Insurance*.

The results indicate that women are more likely than men to visit the PHC, but there was no statistical difference. There was a significant difference in the average utilization of PHC between urban and rural residents: rural residents (average PHC visits = 2.05 times) were more active in PHC utilization compared to urban residents (average PHC visits = 1.56 times). PHC utilization was reduced as the education years increased. For example, respondents with primary education had an average number of visited times of 2.75 in the past year, while those who had graduate degrees visited 1.24 times on an average. In addition, the type of medical insurance can also affect their PHC utilization. The result indicated that the respondents insured by NCMS have higher mean PHC utilization, while the respondents insured by UEBMI had a lower mean PHC utilization (see Table 2).

**Table 2:** Descriptive statistics of variables used in the analysis.

**Variable Category**	**PHC utilization**	***T* test/*F* test**
	**Mean**	**S.D**.	
Gender			*t* = −1.37, *p* > 0.05
Male	1.69	2.91	
Female	1.81	3.27	
Household registration			*t* = 5.68, *p* < 0.05
Rural	2.05	3.12	
Urban	1.56	3.10	
Education			*F* = 21.24, *P* < 0.05
Elementary school or lower	2.75	4.46	
Junior high school	2.02	3.60	
High school	1.85	3.07	
Junior college	1.65	3.11	
Undergraduate	1.31	2.21	
Graduate student or higher	1.24	1.75	
Self-health grade			*F* = 75.08, *P* < 0.05
Very good	3.57	5.44	
Good	3.84	6.06	
Fair	2	3.06	
Poor	1.35	2.38	
Very poor	0.92	1.75	
Healthcare insurance			*F* = 9.59, *P* < 0.05
UEBMI	1.65	3.43	
URBMI	1.69	2.87	
NCMS	2.06	3.24	
None	1.19	2.15	
Spouse occupations			*F* = 10.65, *P* < 0.05
Official	1.76	2.80	
Teacher	1.73	2.51	
Manager	1.34	1.96	
Worker	1.68	2.69	
Farmer	2.58	3.73	
Self-employed entrepreneur	1.63	2.48	
Others	1.42	2.70	
None	1.95	4.33	

The results of the multilevel regression analysis of factors associated with PHC utilization for the overall population are presented in Table 3. The interclass correlation coefficient was lower than 0.4 and the likelihood ratio vs. binomial model in Model 4 was statistically significant, which indicated the appropriateness of using a multilevel model.

**Table 3:** Multilevel regression analysis of factors associated with PHC utilization.

**PHC utilization**	**Model 1**		**Model 2**		**Model 3**		**Model 4**	
	**IRR**	**95%CI**	**IRR**	**95%CI**	**IRR**	**95%CI**	**IRR**	**95%CI**
**Fixed effect part**								
Household registration			0.9725	[0.8541, 1.1072]			0.9721	[0.8538, 1.1067]
Perceived community size			1				1	
Small			0.9884	[0.8512, 1.1477]			0.9990	[0.8527, 1.1494]
Fair			1.0157	[0.8877, 1.1621]			1.0206	[0.8921, 1.1675]
Large			1.0402	[0.8818, 1.2269]			1.0451	[0.8864, 1.2324]
Very large			1.1263	[0.8726, 1.4538]			1.1425	[0.8848, 1.4754]
Gender			1.1948^***^	[1.1085, 1.2879]			1.1944^***^	[1.1081, 1.2874]
Education			1				1	
Junior high school			1.0031	[0.8808, 1.1424]			1.0012	[0.8791, 1.1402]
High school			0.9797	[0.8506, 1.1283]			0.9779	[0.8491, 1.1262]
Junior college			0.8879	[0.7558, 1.0430]			0.8841	[0.7526, 1.0385]
Undergraduate			0.8213^*^	[0.7019, 0.9609]			0.8171^**^	[0.6984, 0.9559]
Graduate student or higher			0.7719	[0.5944, 1.0025]			0.7670^**^	[0.5906, 0.9961]
Spouse occupations			1				1	
Teacher			0.9626	[0.8143, 1.1380]			0.9610	[0.8131, 1.1358]
Manager			0.8358^*^	[0.7021, 0.9950]			0.8341^**^	[0.7007, 0.9929]
Worker			0.7098^***^	[0.6038, 0.8343]			0.7079^***^	[0.6024, 0.8321]
Farmer			0.9057	[0.7548, 1.0868]			0.9014	[0.7515, 1.0813]
Self-employed entrepreneur			0.8499^*^	[0.7301, 0.9893]			0.8481^**^	[0.7287, 0.9872]
Others			0.8594	[0.7254 ,1.0182]			0.8591^*^	[0.7252, 1.0177]
No occupation			0.8438	[0.7093, 1.0039]			0.8432^*^	[0.7088, 1.0030]
Without spouse			0.8139^**^	[0.7071, 0.9367]			0.8150^***^	[0.7082, 0.9380]
Individual annual non-medical expenditures			0.9999^*^	[0.9999, 0.9999]			0.999999491^*^	[0.99999898, 1.000000002]
Years lived at current location			1				1	
1–3 years			0.8510	[0.7013, 1.0327]			0.8524	[0.7024, 1.0343]
4–5 years			0.8842	[0.7216, 1.0833]			0.8841	[0.7216, 1.0832]
6–10 years			0.7742^*^	[0.6292, 0.9525]			0.7759^**^	[0.6306, 0.9545]
11–19 years			0.8339	[0.6713, 1.0360]			0.8366	[0.6735, 1.0392]
≥20 years			0.8939	[0.7225, 1.1059]			0.8931	[0.7221, 1.1047]
Renting housing			1.1190	[0.9932, 1.2606]			1.1200^*^	[0.9941, 1.2619]
BMI			1.0043	[0.9922, 1.0165]			1.0042	[0.9921, 1.0164]
Healthcare Insurance			1				1	
UEBMI			0.9890	[0.8925, 1.0959]			0.9904	[0.8938, 1.0974]
URBMI			0.9708	[0.8312, 1.1340]			0.9670	[0.8279, 1.1295]
NCMS			0.8040^*^	[0.6621, 0.9762]			0.8032^**^	[0.6614, 0.9753]
CMI			1.0004	[0.9169, 1.0916]			0.9990	[0.9157, 1.0899]
Smoke			1.0691	[0.9926, 1.1514]			1.070^*^	[0.9935, 1.1524]
Self-health grade			1				1	
Good			1.0373	[0.7663, 1.4040]			1.0390	[0.7677, 1.4061]
Fair			0.7226^*^	[0.5408, 0.9655]			1.0206^**^	[0.8921, 1.1675]
Poor			0.5144^***^	[0.3832, 0.6907]			0.5166^***^	[0.3848, 0.6934]
Very poor			0.3744^***^	[0.2743, 0.5111]			0.3762^***^	[0.2756, 0.5135]
CCP membership			1.0036	[0.9076, 1.1099]			1.0023	[0.9064, 1.1083]
Chronic number			1.2921^***^	[1.2336, 1.3533]			1.2936^***^	[1.2350, 1.3550]
CSC					1.0282^**^	[1.0095, 1.0472]	1.019^**^	[1.0001, 1.0382]
ISC			1.0046^*^	[1.0001, 1.0092]			1.0035	[0.9988, 1.0083]
_cons	1.3113^***^	[1.18423, 1.4524]	2.1073^***^	[1.3684, 3.2452]	1.4149^***^	[1.2609, 1.5877]	2.106^***^	[1.3680,3.2421]
**Random effect part**								
Community var(CSC)					0.0008	[0.0008, 0.0008]	0.0010	[0.0001,0.0129]
Community var(_cons)	0.3730	[0.0029, 47.9143]	0.5439^***^	[1.5584, 189851]	0.4943	[0.4943,0.4943]	0.3508	[4.2204E-12, 2.9164E+10]
Community cov(_cons, CSC)					−0.0202^***^	[−0.020, −0.020]	−0.009	[−0.0205,0.0026]
Community>city var(_cons)	0.2843	[0.0005, 167.2552]	0.0836^***^	[2.4713, 2.8328]	0.341892182	convergence not achieved	0.2354	[1.2502E-17, 4.4312E+15]

*CI, Confidence Interval*.

* *p ≤ 0.1*

** *p ≤ 0.05*

*** *p ≤ 0.001. interclass correlation coefficient of dependent variable = 0.1649*.

*All variables, except for the community indicator, are centered on their grand mean. This approach reduces multicollinearity, facilitates interpretation of the intercept, and does not affect the interpretation of the covariates in the model. a - grand mean centered. This table reports unstandardized coefficients that form a hierarchical binomial regression (Population-average model with robust standard errors). In the parentheses, we report the odds ratios for a one standard deviation change in the predictor variable. model 4- LR test vs. nbinomial model: chi2(4) = 957.01 Prob > chi2 = 0.0000*.

The results of Model 2 show that if the community level variable was not included in the model, all individual-level variables were significantly associated with PHC utilization, except household registration, perceived community size, BMI, and ISC.

The results of Model 3 indicate that if only CSC in the community level was included in the model, there were interaction effects between the two levels but not statistically significant (*p* > 0.1). However, the convergence of the third level was not achieved possibly due to limited iterations.

In Model 4, the incidence-rate ratios (IRR) of each variable did not change significantly (Table 3) compared to Model 2 and Model 3 when all individual and community variables were included in the analysis, and only CSC had a statistical significant association with the dependent variable that one standard deviation increase in the CSC was associated with a 1.9% increment in PHC utilization (*p* < 0.05). Additionally, many other variables included in the model were significantly associated with the PHC utilization of the respondents, and among them, self-health state of “very poor” or “poor” and the occupation being worker had the largest negative association with PHC utilization, and the number of chronic diseases, gender being female, and perceived community size being very large had the largest positive association.

## Discussion

This study investigated the association of social capital with PHC utilization in China applying the concept of individual-level and community-level effects. The results indicated that CSC has a positive association with PHC utilization, while the association of ISC with PHC utilization is not significant. Some of the covariates, such as gender and education, also influenced PHC utilization. This study provided a comprehensive new perspective for research in the field of PHC utilization in developing countries, and also supported the following discussion.

This research was carried out in China, an Asian country with a cultural background that values close social relationships like kinship and neighborhood, and it is easier for local residents to obtain social capital and where residents are more inclined to use social capital to perform healthy behaviors. Previous studies have shown that social capital could be influenced by cultural background, and social capital under East Asian culture functions differently compared to Western society (54). For example, in Chinese traditional agricultural society, blood- and kinship-related networks are common social-capital carriers (social capital works through certain social carriers). In modern Chinese society, there exist a large number of civic organizations that have provided a form of Chinese social capital based on a small-size peasant economy and regulated by traditional customs and patriarchal systems. Social capital in this culture can provide a sense of security, trust, and identity to residents, which accelerates the generation and accumulation of trust between Chinese residents. This social capital in turn makes social capital more important in the collectivist countries represented by China, and it deserves deep exploration. Above all, social capital in Chinese culture makes it difficult for Chinese residents to choose resources (including health resources) in an unfamiliar way. If people in social networks tend to use PHC services, each individual in this social network will be more willing to choose PHC institutions and vice versa, which can be an interpretation of our research results.

The findings of this study could not support the hypothesis that ISC is positively associated with PHC utilization. This may be due to that though ISC increases patient trust in PHC providers, which helps to build a good physician-patient relationship (55), in China, this mechanism may work on the relationships of the residents with the health care providers in both the PHCs and the secondary/tertiary hospitals. Moreover, ISC in the form of neighbors and friends can motivate groups to implement and maintain social norms (i.e., informal social controls) (56), but in China, preference of the residents for PHC and the secondary/tertiary hospitals seems to be equal, and no clear social norm has yet been formed (57).

Against the second hypothesis in this study, the results of this study show that residents living in communities with higher CSC have a higher PHC utilization, which is different from previous studies: the association of CSC with PHC utilization is not always positive (17). The results of this study may be explained as follows: unlike ISC, which covers a wide range of social relations, CSC is limited within the community a resident lives in, which potentially makes ISC and CSC associated with the PHC utilization different. CSC is very important for generating mutual aid and protecting the vulnerable as well as improving the trust of the residents and dependence on the community and surrounding public facilities (including PHC institutions), which improves PHC utilization (58). These results are consistent with the theoretical role of social capital in improving the efficiency of health services (59).

These two findings are not necessarily contradictory because each form of social capital may operate through different mechanisms: ISC may improve access between inefficient users (i.e., transportation services), while CSC might serve as a substitute for PHC utilization that might involve counseling/caring services (49).

In summary, this study suggests that social capital may play an important role in improving PHC utilization, although the mechanisms need to be identified by further research. If social capital does contribute to more efficient utilization of PHC, then the question is how to manipulate social capital to achieve this goal.

Some covariates have great significance in explaining PHC utilization. First, unlike previous studies (49), this study indicated that age and community size have no significant association with the utilization of PHC. We think that this lack of association may be due to the fact that the demand for care is usually more complex according to the age increments, so there are uncertainties about PHC utilization among different age groups. Although the association of community size with PHC utilization is not significant, the regression results show that there is a tendency for residents of larger communities to have higher PHC utilization. One possible explanation is that communities with a higher population size have higher social activity than smaller communities, and in larger communities, residents are committed to solving problems through community activities and community participation, such as developing community health services and health education, which can improve the utilization of PHC services of residents (60).

Second, higher education background of the respondents was significantly correlated with lower PHC utilization, which supports the argument by Grossman that educated people are generally healthier and need less access to health services, including PHC services (61). Income is another important factor in PHC utilization. Relative income (income centralized at the community level) was used in this study because it captures the community-level variation more sensitively, and this variation explains the improvement or reduction in quality of life for individuals with similar incomes caused by the differences among communities. In this study, there was a negative correlation between income and PHC utilization, which indicates that the health services provided by PHC institutions are not attractive to patients with high purchasing power that is inconsistent with the findings of Doorslaer and Masseria (62). Additionally, Makinen et al. (63) found that high earnings lead to higher use of health services in developing countries. This mechanism seems to only apply to secondary and tertiary hospitals rather than PHC institutions because higher payment capacities provide more choices for different health institutions, and the priority for the choice of residents for health care setting would be based on the safety and quality of medical care instead of price. Under the current health system in China, tertiary hospitals may be the best option for high-income groups. They do not tend to choose PHC institutions with lower prices that have relatively unsatisfying quality.

Primary health care utilization also varies among respondents with different types of health insurance. Generally speaking, residents with health care insurance have lower PHC utilization than individuals who are uninsured because the insurances improved the affordability of health care to the insured residents. For residents insured by UEBMI or URBMI, their PHC utilization is higher than those insured by NCMS. The reason may be the differences in the financing mechanism, scope of reimbursement, and payment standards of the above three types of health care insurance in China (64). For example, the higher reimbursement rate of NCMS increases the health service needs of higher quality health care among insured rural residents. This compensation mechanism facilitates the intention of the rural residents of visiting secondary or tertiary hospitals. A previous study showed that NCMS increased the health services utilization for Chinese rural residents, which is consistent with the results of this study (65). Those insured by UEBMI usually have a higher level of occupational stress, leading to limited time and energy for seeking health care, which could be the main driver of the residents for utilizing the PHC. Most of the residents covered by URBMI are retired, who have adequate time but are limited in energy for visiting secondary or tertiary hospitals, which are more distant from the residential areas than PHC institutions. Also, their higher prevalence of chronic or mild diseases may increase their utilization of PHC, which is designed for those health care needs.

In addition to the confounders mentioned above, social capital helps to create a more effective and better-coordinated PHC system where health care institutions and general practitioner (GP) can provide better health services, and patients might be more likely to visit PHC institutions because of their trust.

Although the results of this study are the best approximation of these relationships, some limitations have to be considered. First, to control the length of the questionnaire, the study did not consider the cofounders of city-level (or those of levels higher than a city) PHC utilization, including the regional culture and policies of the provinces. For example, in the context of the National Hierarchical Medical System of China, health policies vary among different provinces, which brought about the differences in PHC utilization of residents. Second, this study did not consider the stock characteristics of social capital. We have not measured the relationship between social capital and PHC utilization over a period of time, so there might be bias caused by time factors that arise due to the cross-sectional characteristics of the data. The causality between PHC utilization and social capital over time could not be determined. Third, Model 3 did not achieve the convergence based on the maximum iterations the researcher was able to carry out, and it is possibly because that the variance of the first level was included in the second and third levels when no independent variable was added in the first level. One of the strengths of this study is the use of nationwide survey data, but it also indicates that the variables available for analysis are limited because to retain a relatively large sample and control the quality of the data, this survey did not cover all potential control variables.

## Conclusions

This study suggests that in China, CSC may play an active role in improving PHC utilization, but ISC has no significant association with PHC utilization, indicating that social capital plays a major role at the community level and has significant spillover effects in China. It also suggests constructing and improving CSC from the perspective of generating a positive association with PHC utilization by individuals, families, and neighbors in the community. The overarching policy implication from the results of this study is that there is a meaningful interplay between PHC utilization and social capital that ought to be considered in health policy and planning in China. Future research should focus on how social capital affects PHC utilization over time.

## Data Availability Statement

The raw data supporting the conclusions of this article will be made available by the authors, without undue reservation.

## Ethics Statement

The ethical approval to conduct the pilot survey and main survey was granted by the Ethics Committee of China Pharmaceutical University (Project Number: CPU2018009). Written consent to participate was obtained from all participants.

## Author Contributions

WZ and XX contributed to the conception and design of the study. WZ and YH contributed to the drafting of the study. WZ, YH, and TW contributed to the analysis, interpretation of data, and the revision of the study. ML, GL, and TW contributed to the acquisition and analysis of data. WZ, YH, and XX contributed to the interpretation of data. All authors had read and approved the final version of the manuscript for submission.

## Conflict of Interest

The authors declare that the research was conducted in the absence of any commercial or financial relationships that could be construed as a potential conflict of interest.

## Publisher's Note

All claims expressed in this article are solely those of the authors and do not necessarily represent those of their affiliated organizations, or those of the publisher, the editors and the reviewers. Any product that may be evaluated in this article, or claim that may be made by its manufacturer, is not guaranteed or endorsed by the publisher.
