# Supplementary material for: I Know Some People: The Association of Social Capital With Primary Health Care Utilization of Residents in China
Source: Front Public Health. 2021 Jul 30;9:689765. doi: 10.3389/fpubh.2021.689765 (PMC8360841; doi:10.3389/fpubh.2021.689765)
Supplement: Supplementary file 1 [file Data_Sheet_1.docx]

**Questionnaire**

1. Name of the respondent's neighbourhood__________________
2. How do you feel about the size of your current neighbourhood?

A. Small scale B. Smaller scale C. General D. Larger scale E. Large scale

1. Your gender is

A. Men B. Women

1. Your age is ___________________.
2. Are you a member of the Chinese Communist Party?

A. Yes B. No

1. Your account (resident) type is

A. Rural B. Urban

1. Your level of education is

A. Primary school and below B. Middle school C. High school or junior high school D. College F. Bachelor's degree G. Master's degree and above

1. Your spouse's occupation is

A.Public officials (including civil servants and persons in institutions other than teachers) B.Teachers C.Business managers D.Workers (including migrant workers) E.Farmers F.Self-employed G.Other H.No work I.Currently no spouse

1. Your personal annual income is approximately (in dollars, please) ___________________
2. The number of years you have lived in your current community is

A.1 years or less B.1-3 years C.4-5 years D.6-10 years E.11-19 years F.20 years and above

1. Is the house you currently live in an owned property or a rental?

A. rented house B. own property

1. What is your height _____ (cm)?
2. What is your body weight in kilograms (kg)?
3. Which of the following basic types of health insurance do you currently have?

A. Medical insurance for urban workers B. Medical insurance for urban residents C. New rural cooperative medical care D. No such medical insurance

1. Do you have commercial health insurance?

A.No B.Yes

1. Do you or your family smoke?

A. No B. Yes

1. How do you consider your health to be?

A.Poor B.Poorer C.Average D.Better E.Good

1. In the past 1 year, you have visited several community hospitals (please fill in specific numbers) ______
2. Which of the following chronic conditions do you currently suffer from (multiple choices available)

A.Cerebrovascular disease B.Heart failure C.Heart disease D.Asthma E.Chronic obstructive pulmonary disease F.Diabetes G.Chronic hepatitis H.Chronic kidney disease I.Arthritis J.Hypertension K.Osteoporosis L.Periodontal disease M.Thyroid disease N.Cancer O.Other P.No chronic disease

In every community, some people get along with others and trust each other, while other people do not. Now, I would like to talk to you about trust and solidarity in your community.

1. Generally speaking, would you say that most people can be trusted, or that you can’t be too careful in your dealings with other people?

A.Most people can be trusted B.You can’t be too careful

1. In general, do you agree or disagree with the following statements?
2. Most people who live in this village/neighborhood can be trusted.

A.Agree strongly B.Agree somewhat C.Neither agree nor disagree D.Disagree somewhat E.Disagree strongly

1. In this village/neighborhood, one has to be alert or someone is likely to take advantage of you.

A.Agree strongly B.Agree somewhat C.Neither agree nor disagree D.Disagree somewhat E.Disagree strongly

1. Most people in this village/neighborhood are willing to help if you need it.

A.Agree strongly B.Agree somewhat C.Neither agree nor disagree D.Disagree somewhat E.Disagree strongly

1. In this village/neighborhood, people generally do not trust each other in matters of lending and borrowing money.

A.Agree strongly B.Agree somewhat C.Neither agree nor disagree D.Disagree somewhat E.Disagree strongly

1. Now I want to ask you how much you trust different types of people. On a scale of 1 to 5, where 1 means a very small extent and 5 means a very great extent, how much do you trust the people in that category?
2. People from your ethnic or linguistic group/race/caste/tribe

A.To a very small extent B.To a small extent C.Neither small nor great extent D.To a great extent E.To a very great extent

1. People from other ethnic or linguistic groups/race/caste/tribe

A.To a very small extent B.To a small extent C.Neither small nor great extent D.To a great extent E.To a very great extent

1. Shopkeepers

A.To a very small extent B.To a small extent C.Neither small nor great extent D.To a great extent E.To a very great extent

1. Local government officials

A.To a very small extent B.To a small extent C.Neither small nor great extent D.To a great extent E.To a very great extent

1. Central government officials

A.To a very small extent B.To a small extent C.Neither small nor great extent D.To a great extent E.To a very great extent

1. Police

A.To a very small extent B.To a small extent C.Neither small nor great extent D.To a great extent E.To a very great extent

1. Teachers

A.To a very small extent B.To a small extent C.Neither small nor great extent D.To a great extent E.To a very great extent

1. Nurses and doctors

A.To a very small extent B.To a small extent C.Neither small nor great extent D.To a great extent E.To a very great extent

1. Strangers

A.To a very small extent B.To a small extent C.Neither small nor great extent D.To a great extent E.To a very great extent

1. Do you think that over the last five years, the level of trust in this village/neighborhood has gotten better, worse, or stayed about the same?

A.Gotten better B.Gotten worse C.Stayed about the same

1. How well do people in your village/neighborhood help each other out these days? Use a five point scale, where 1 means always helping and 5 means never helping.

A.Always helping B.Helping most of the time C.Helping sometimes D.Rarely helping E.Never helping

1. If a community project does not directly benefit you, but has benefits for many others in the village/neighborhood, to what extent would you contribute time to the project?

A.To a very small extent B.To a small extent C.Neither small nor great extent D.To a great extent E.To a very great extent

1. If a community project does not directly benefit you, but has benefits for many others in the village/neighborhood, to what extent would you contribute money to the project?

A.To a very small extent B.To a small extent C.Neither small nor great extent D.To a great extent E.To a very great extent
